# Prediction of the caved rock zones’ scope induced by caving mining method

**DOI:** 10.1371/journal.pone.0202221

**Published:** 2018-08-15

**Authors:** Fengyu Ren, Yang Liu, Jianli Cao, Rongxing He, Yu Fu, Yanjun Zhou, Huan Liu

**Affiliations:** School of Resources and Civil Engineering, Northeastern University, Shenyang, Liaoning Province, China; Politecnico di Milano, ITALY

## Abstract

Critical medium column theory has typically been used to predict the scope of caved rock zone (CRZ) caused by caving mining method. It is essential to understand the distribution laws of lateral pressure induced by caving mining method with different dipping angles. In this study, a self-designed scaled physical model was used to investigate the distribution laws with angles ranging from 80° to 90°, and ore drawing was employed in experiments to simulate caving mining method. The experimental results indicated that the distribution laws were divided into the reductive region and the extensive region during ore drawing. The reductive region was close to the drawing hole, and its scope was smaller than the other part. Moreover, decreasing the dipping angle was an effective way of controlling the maximum reduction rates and the scope of reductive region. By varying laws of lateral pressure, the predictive model of CRZs’ scope was established. Additionally, the rock mass located outside the predictive CRZs’ scope did not rupture based on the monitoring of Digital Optical Televiewing (OPTV), which was verified by the field test in Dabeishan Iron Mine, China. The results of the field test demonstrated that the prediction method used in this study was valid and could be used in practice.

## Introduction

Caving mining method has been widely used in metal mines around the world. Approximately 25% of mines worldwide use this method [1]. Also, more than 85% of iron ores and approximately 40% of non-ferrous metal ores in underground mines are mined out using this method in China [2]. A characteristic of caving mining method is caving and extraction of the massive volume of ore, which consequently induced a cave rock zone (CRZ) in the vicinity of the mining operation. An uncontrolled CRZ can endanger mine exploitation and is a major concern for operational safety. Furthermore, the inappropriate prediction of CRZ will result in wasting mineral resources and decreasing economic benefits of mining enterprises [3, 4]. Hence, a large number of research studies have focused on the predicting the scope of CRZ.

To date, conventional laws have been based on the theory of Van As and the critical medium column theory [5, 6]. The theory of Van As is primarily based on using a certain angle of break to delineate the scope of CRZ and is widely used in coal mines. Currently, mining up to depth of 1,000 m, or even 2,000 m below the ground surface, is a very common practice in metal mines [7–9]. However, this theory predicts the scope of CRZ possessing a large drawback in the deep metal mines. The critical medium column theory introduces the concept of the critical medium column, in which a critical height exists in the CRZ. The lateral pressure of the filling medium can prevent rock failure in the CRZ when the height of the filling medium is higher than the critical height. Consequently, the scope of CRZ remains constant with the increase of mining depth. Thus, this theory has been widely used in metal mines, such as Gongchangling Iron Mine and Xizhimen Iron Mine in China [10]. With the application of an inappropriate lateral pressures’ distribution, the safety indices can significantly deteriorate. Therefore, the distribution of lateral pressure has a large effect on controlling and avoiding the extension of a CRZ during the mining process.

In situ trials could better reflect the full similitude of actual mining conditions. Due to the high cost, time consumption, and practical difficulties associated with directly managing and observing the in situ waste rock filling, the present investigation for the bulk solids’ mechanics was usually achieved through laboratory experiments. Scaled physical models have been widely used in laboratory settings, and such experiments have been critical in studies of the problems associated with lateral pressure. For instance, the pressure coefficients and the equilibrium states’ mechanics were studied in experiments conducted by researchers using rock, sandy soil, and aggregate as materials. Some main influencing factors have been studied, such as the pressure coefficients, the mechanical mechanism under the equilibrium state, the characteristics of active earth pressure and passive earth pressure. Moreover, many laboratory granule experiments were conducted using scanning electron microscope (SEM) to provide more insight into the varying laws of bulk solids [11–14], which have promoted research development in this field.

With advances in computing technology, many numerical codes, including 3DEC [15], PFC3D [16], DDA [17] and ELFEN [18], were developed and applied to ground subsidence modelling. FLAC3D software was employed to analyse the safe roof thickness, stress, deformation, and plastic zones of the gob area after excavation [19]. The numerical code RFPA2D was applied to generate surface subsidence profiles and failure modes at different initial caving depths [20]. However, these models have been usually conducted to verify mine safety.

The distribution laws of lateral pressure are closely related to the motion state of bulk solids, which has also been a topic of considerable research interest in the field of the lateral pressure [21, 22]. In caving mining method, the lateral pressures’ distribution could be influenced by mining in the lower orebody. However, most studies have aimed to simulate the lateral pressures’ distribution under the limiting equilibrium state. Few studies on this distribution laws under ore drawing conditions have been conducted thus far. Although some important factors, such as the particle size, the properties of bulk solids and the type of CRZ, have an impact on the distribution laws, ore drawing is also a critical factor in the successful prediction of the scope of CRZ. Considering the influence for ore drawing on the distribution laws, scaled physical experiments with different dipping angles were carried out. Then, the characteristics of the incidence range and variation trends for lateral pressure were obtained, and the prediction method of the CRZs’ scope was modified. Finally, the modified formula was verified by the field test in Dabeishan Iron Mine, China. The proposed prediction formula can play an important role in mining design and production management.

## Methodology for calculating the scope of a CRZ

**Fig 1:**
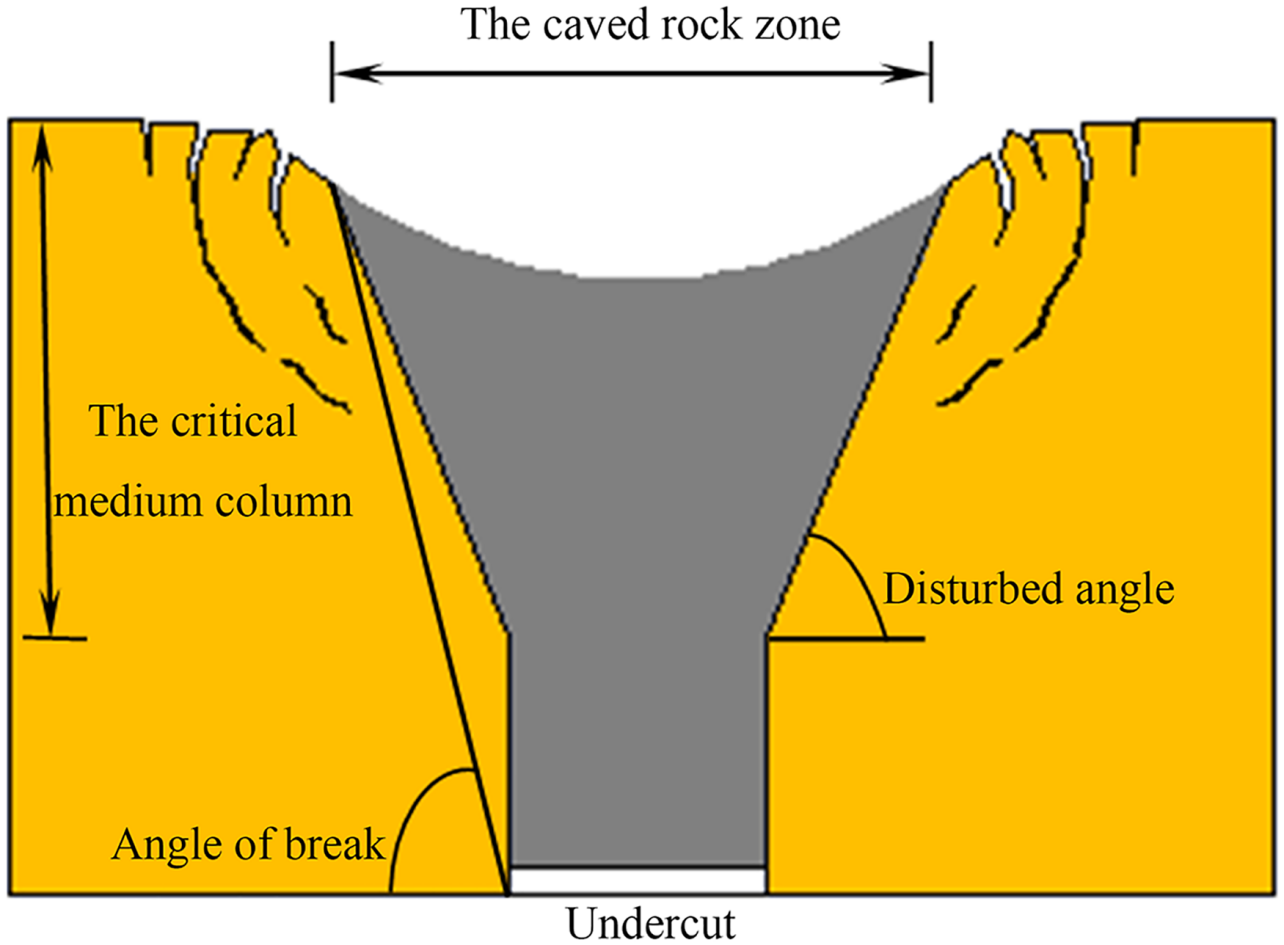
Sketch map of the caved rock zone and the disturbed angle.

The critical medium column theory is proposed to predict the scope of CRZ induced by caving mining method in metal mines. A schematic figure of a CRZ is shown in Fig 1, the scope of CRZ is raised as follows [7]:

$$L=2H_{0}\text{cot}\beta +L_{t}$$ (1)

where *H*_*0*_ is the height of the critical medium column, (m); *β* is the disturbed angle, (°); *L* is the scope of the CRZ, (m); and *L*_*t*_ is the length of gob, (m).

*H*_*0*_ is essential predicting the scope of CRZ using the critical medium column theory. According to the classical Janssen bulk solid pressure theory [23, 24], the equation of lateral pressures under the limiting equilibrium state is described as follows:

$$p_{s}=\frac{\gamma S}{fC}\text{sin}\alpha \left(1-\frac{f}{\text{tan}\alpha }\right)\left[1-e^{-\frac{fKC}{S\text{sin}\alpha }z}\right]$$ (2)

where *γ* is the unit weight of bulk solids, (N·m^-3^); *z* is the depth of bulk solids, (m); *f* is the friction coefficient between bulk solids and the collapse pit wall, (°), *f = tanϕ*, in which *ϕ* is the friction angle between bulk solids and the collapse pit wall; *K* is the coefficient of bulk solids’ lateral pressures, $K=\frac{1-\text{sin}\theta }{1+\text{sin}\theta }$, in which *θ* is the internal friction angle of bulk solids, (°); *p*_*s*_ is the lateral pressure of bulk solids, (Pa); *α* is the dipping angle, (°); *S* is the horizontally projected area of the CRZ, (m^2^); and *C* is the horizontally projected circumference of the CRZ, (m).

Combining the critical medium column theory and Eq (2), the predicting formula of the scope of CRZ can be simplified as follows:

$$L_{}=L_{t}+2H\text{cot}\beta -2\text{sin}\alpha \text{cot}\beta S\frac{\left(\text{ln}\frac{\gamma S\text{sin}\alpha \left(1-\frac{f}{\text{tan}\alpha }\right)}{\gamma S\text{sin}\alpha \left(1-\frac{f}{\text{tan}\alpha }\right)-pfC}\right)}{fKC}$$ (3)

where *p* is the minimum lateral pressure that can prevent rock from failure in the CRZ, (Pa), which is determined based on the mine field survey or numerical simulation analysis; and *H* is the mining depth, (m).

However, previous studies have neglected the influence of caving mining method on the lateral pressure, consequently yielding the inaccurate scope of CRZ. To remedy the above drawback, the ore drawing experiments were conducted, and distribution laws were presented in the following section.

## Physical experiments

### Ethics statement

The study was conducted at Dabeishan Iron Mine in Liaoning Province, China (41.22 degrees north latitude and 123.41 degrees east longitude). All necessary permits for the field studies described herein (which did not involve endangered or protected species) were obtained thanks to Tao Su, Director of the Dabeishan Iron Mine Corporation of Benxi (Liaoning Province, China).

### Experimental model and materials

**Fig 2:**
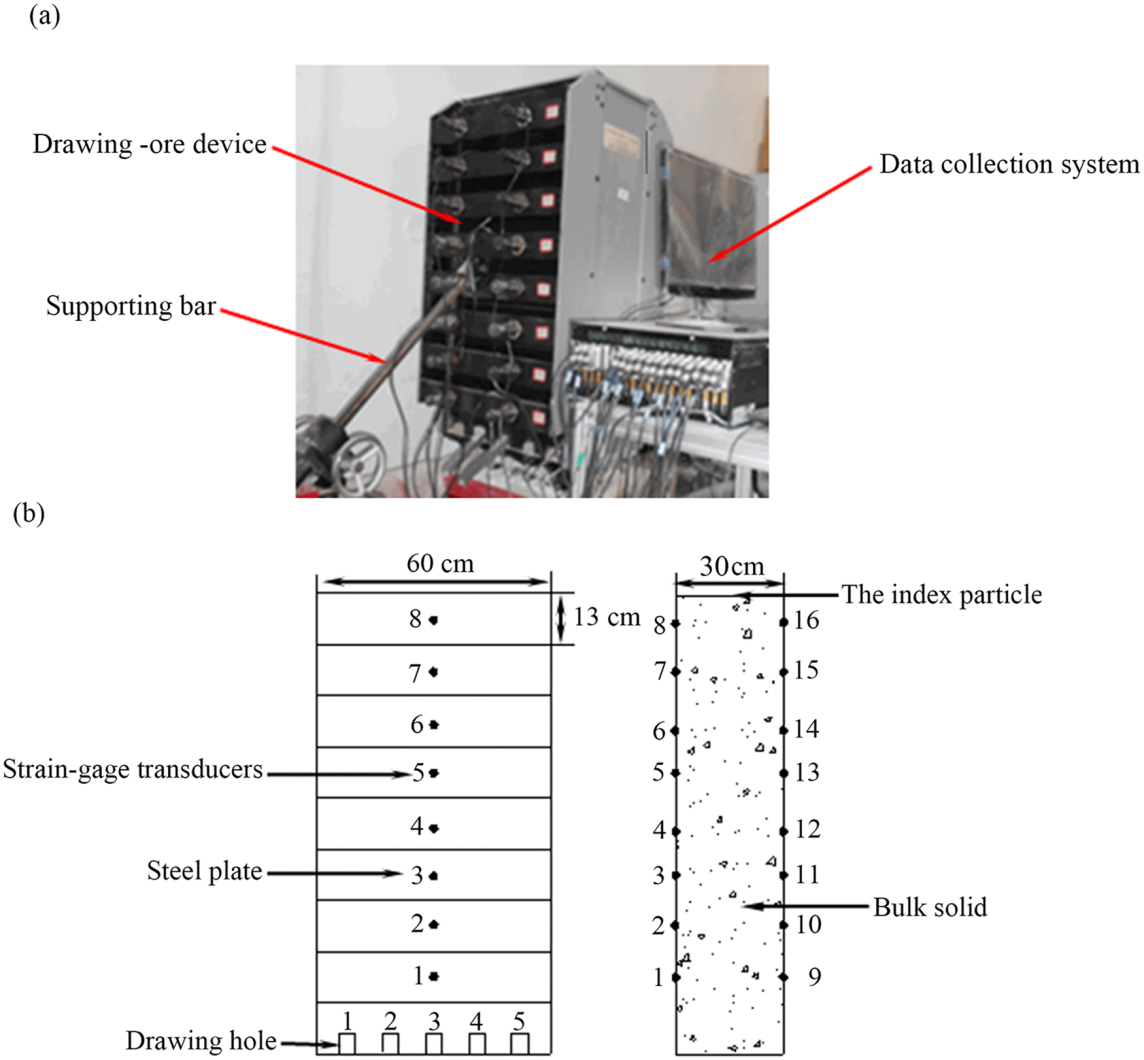
Experimental setup of lateral pressure during ore drawing.

Castro reported that the geometrical scale has no significant influence on the operation or flow capability using a large 3D physical model [25]. Thus, some hypotheses of scaling were raised as follows: (1) The bulk density and the residual friction angle in the model were the same as those in the field. (2) The wall friction angle was similar to the internal friction angle. Based on the similitude principle, a scaled physical model with a geometrical scale of 1:200 was designed to investigate the distribution laws of lateral pressure during caving mining method. As shown in Fig 2, the size of the model was 0.6 m × 0.3 m × 1.17 m (width × length × height). The setup was composed of a drawing-ore device, a data collection system, upper and lower walls, front and back walls, a supporting bar for adjusting the dipping angle, drawing holes and strain-gage transducers. The drawing holes (the width and length were both 0.03 m) were located on the bottom of the lower wall and their separation distance was 0.12 m. The walls were made of steel because bulk solids simulated in these experiments were moderately stable. To accurately measure data at different heights of the bulk solids, the upper and lower walls were disassembled by 16 separate plates, and the strain-gage transducers were located in the centre of each plate. The 1#-8# strain-gage transducers were located in the lower wall, and the 9#-16# strain-gage transducers were located in the upper wall. Furthermore, the 3# drawing hole was used so that the flow zones did not intersect with the walls of model. The bulk solids were adopted the dolomite, its grade was less than 3 mm in the experiments, and its density was 2030 kg·m^-3^. The material was cohesion less and had a friction angle of 34° when tested under conditions similar to those in the model. The calculated model wall friction angle was approximately 30.5°. To illustrate the effect of ore drawing, the dipping angles ranging from 80° to 90° with intervals of 5° were considered.

### Experimental processes

**Fig 3:**
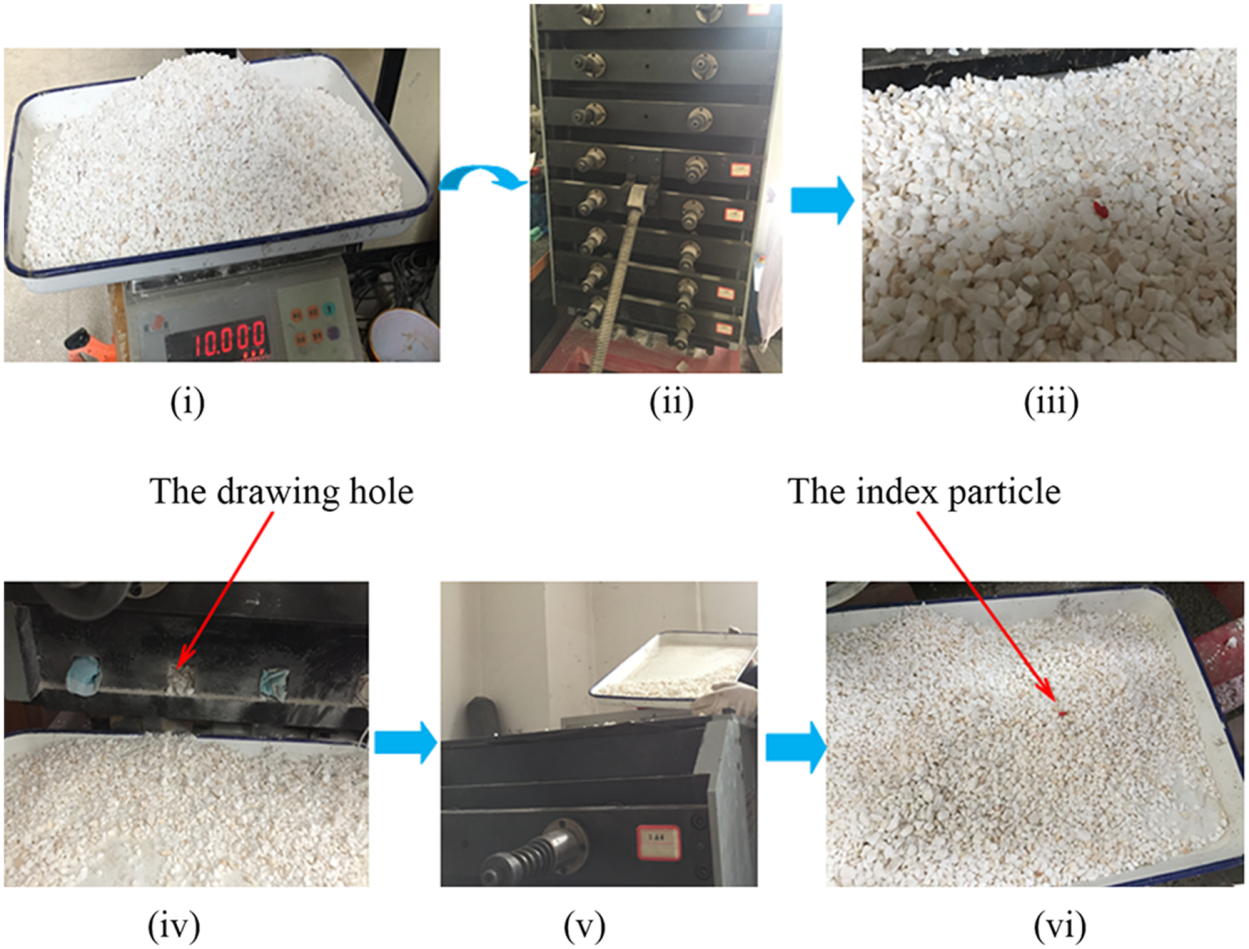
Experimental process. (i) Measured dolomite density; (ii) experimental equipment used for lateral pressure analyses; (iii) dolomite in the experimental equipment with loaded index particles; (iv) drawing of the bulk solids; (v) backfilling the bulk solids; (vi) drawing of the index particle.

Zhang investigated the effects of the ore body dip and width on the characteristics of the flow axis and draw body shape using ore drawing experiments [26]. That study provided the fundamental procedure and theoretical basis for these ore drawing experiments. Three physical experiments were performed to investigate the effects of dipping angle and ore drawing on the distribution laws of lateral pressure. Three different dipping angles (with angles of 80°, 85° and 90°) were taken into account in this study. To reduce the impact of bulk solids’ random movements, each physical simulation scheme was repeated three times. To make the flow characteristics of the index particles consistent with the experimental materials, the index particles were directly selected from the dolomite. Each index particle was sprayed with red paint to distinguish from the experimental materials. The entire model was kept horizontal on the ground to avoid causing opposite effects on the laboratory record. The experimental process as follows (Fig 3): First, the data collection system was linked to the strain-gage transducers and calibrated in initial state. After finishing the calibration, the holes were sealed using elastic materials to simulate the original condition without blasting. Next, the incompact bulk solids completely filled into the device, and the index particles were then located the coordinate points. The relationship between the lateral pressure and the depth of bulk solids was recorded in the initial equilibrium state. Additionally, the total mass of bulk solids was also statistically analysed, and the density of bulk solids was then calculated once the ore loading process was terminated. After that, the elastic material sealing the 3# drawing hole was removed, and then bulk solids were drawn from the drawing hole. During the process of ore drawing, the flow velocity of the bulk solids should not be fast and remain as constant as possible. Meanwhile, the single mass of each draw was calculated, and the lateral pressures with different heights were recorded. To draw bulk solids under a stable pressure and maintain a smooth surface, the incompact bulk solids should be duly added on the surface once the bulk solid draw starts to arrive at the drawing hole. Consequently, the lateral pressures were recorded for each draw until the index particle was drawn, and the distribution laws were then obtained.

## Experimental results and discussion

### The lateral pressure under the limiting equilibrium state

**Fig 4:**
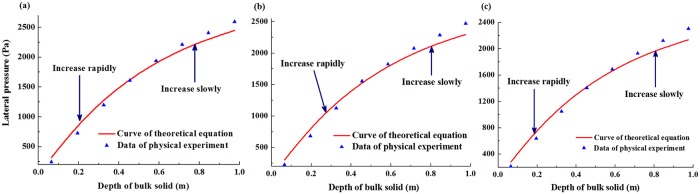
The relationships between the depth of bulk solids and the lateral pressure obtained using physical experiments and theoretical equation with different angles under the limiting equilibrium state. (a) 90°; (b) 85°; (c) 80°.

The relationships between the depth of bulk solids and the lateral pressure obtained using physical experiments and theoretical equation were compared with different angles under the limiting equilibrium state (Fig 4). The varying laws of lateral pressure could be divided into the following two stages: In the initial stage, the lateral pressure increased rapidly, while this growth rate decreased gradually as the depth increased. In the second stage, the value raised slowly as the depth increased. Meanwhile, the growth rate decreased gradually as the dipping angle decreased. This analysis revealed that the values and varying laws in the classical Janssen bulk solid pressure theory were almost the similar as those in the physical experiments. Since the unit weight in Eq (2) was the average value, the unit weight of bulk solids in the lower level was greater than that in the upper level. As a result, the lateral pressure obtained by experiments was greater than that of Eq (2) in the lower level, and the opposite phenomenon occurred in the upper level. The analysis results indicated that Eq (2) represented a good fit with the physical experimental data, and this theory was applied to predict the scope of CRZ under the limiting equilibrium state.

### The lateral pressure during ore drawing

**Fig 5:**
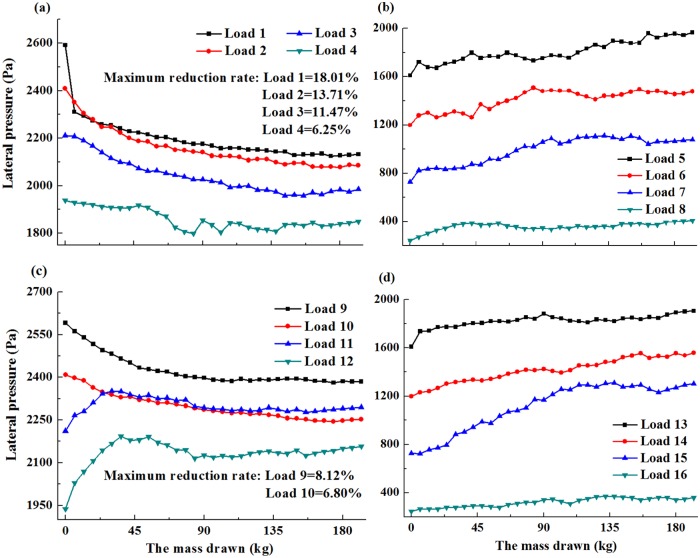
Depth of bulk solids and the lateral pressure with the dipping angle of 90° (a-d).

**Fig 6:**
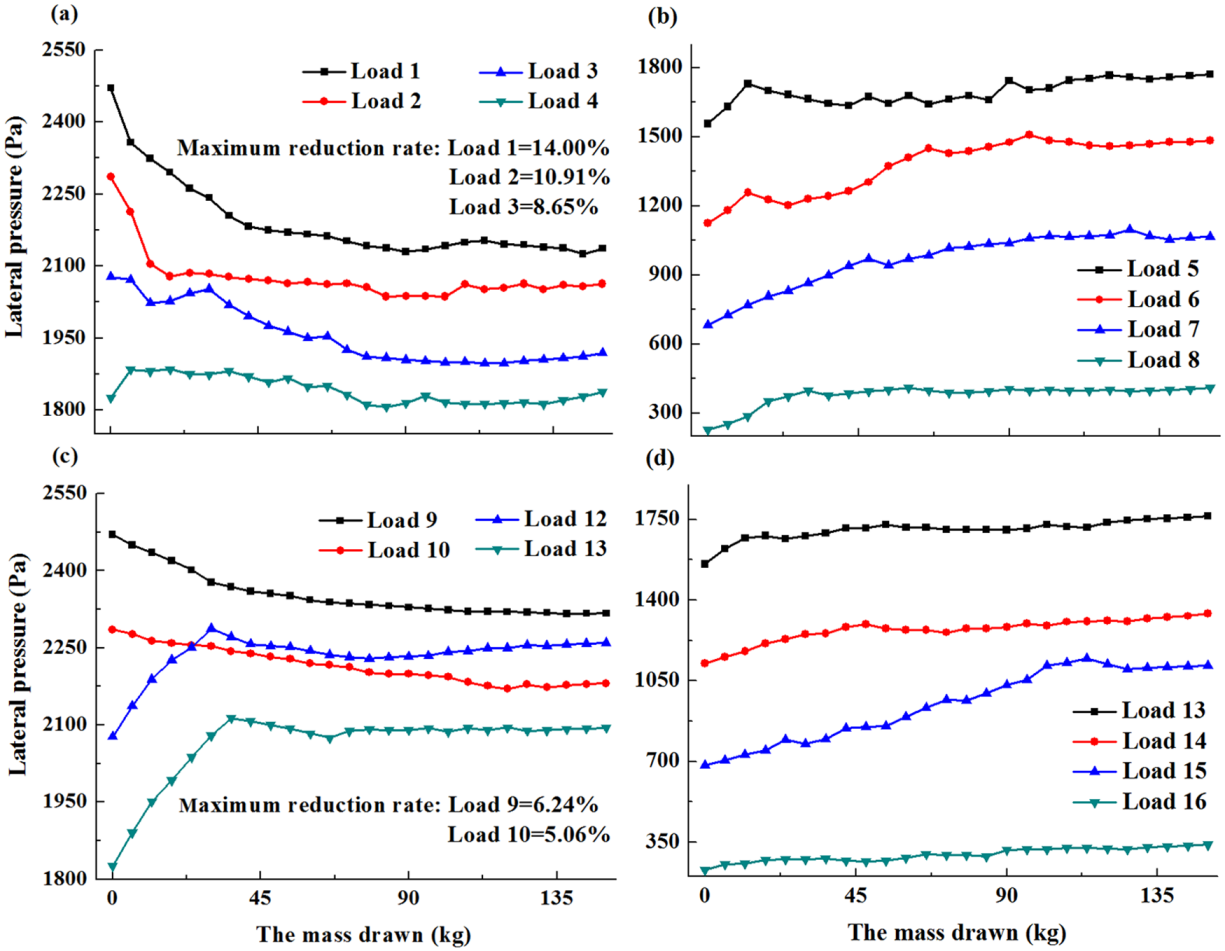
Depth of bulk solids and the lateral pressure with the dipping angle of 85° (a-d).

**Fig 7:**
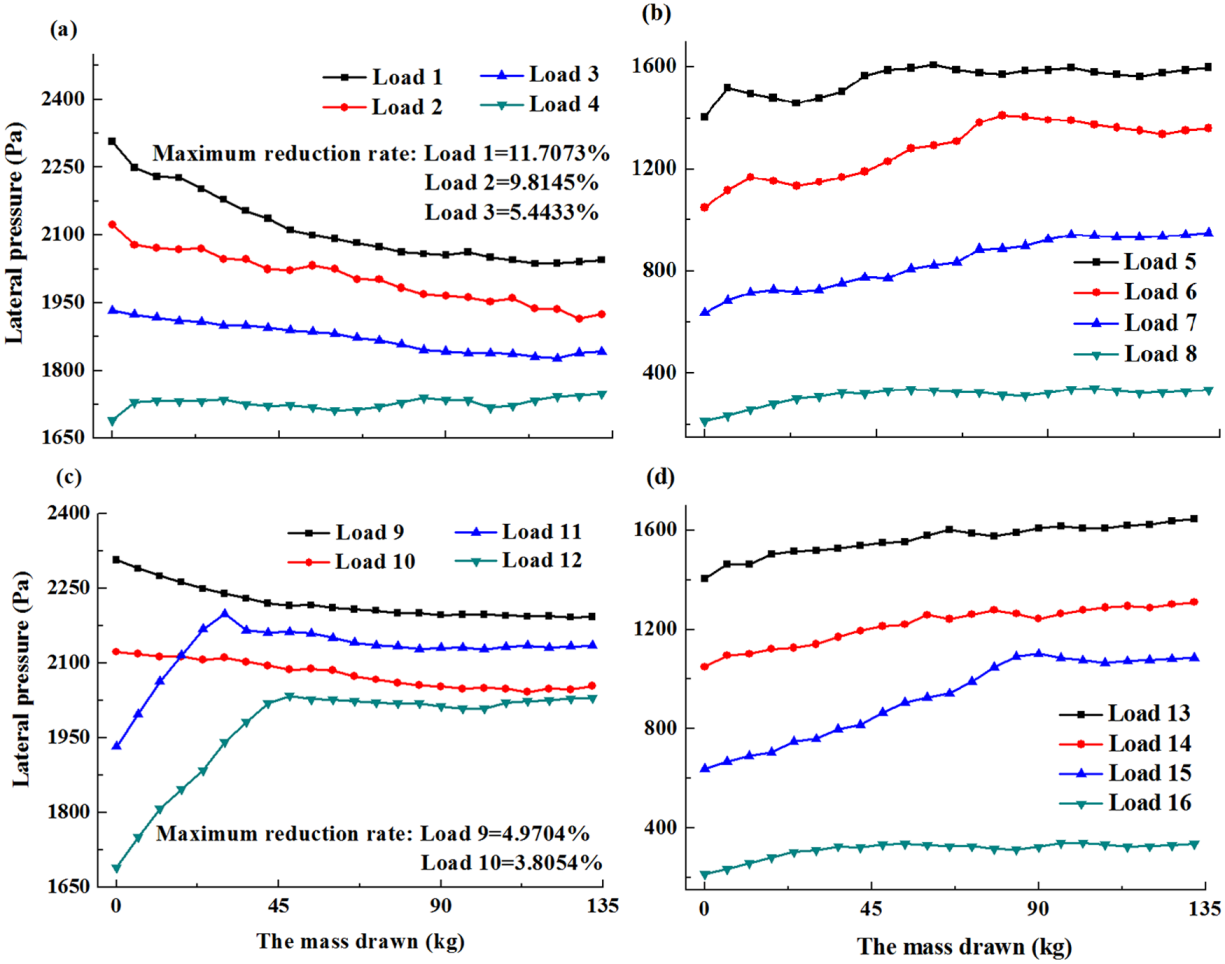
Depth of bulk solids and the lateral pressure with the dipping angle of 80° (a-d).

By weighing the drawn mass and recording the lateral pressure observed with different heights, the distribution laws during ore drawing were clearly obtained. The relationships between the depth of bulk solid and the lateral pressure is possessed with the dipping angles of 90°, 85° and 80°, respectively (Figs 5–7).

In Fig 5, the values of Load 1, Load 2, Load 3, Load 4, Load 9 and Load 10 decreased as the mass drawn increased. These values slightly increased as the mass of the extracted ore continued to increase. The maximum reduction rates of these loads were 18.01%, 13.71%, 11.47%, 6.25%, 8.12% and 6.80%, respectively. Fig 6 showed that the values for Load 4 were relatively similar to those in the limiting equilibrium state with a few small fluctuations. The values for Load 1, Load 2 and Load 3, Load 9 and Load 10 decreased as the mass drawn increased. The maximum reduction rates of these loads were 14.00%, 10.91%, 8.65%, 6.24%, and 5.06%, respectively. For Fig 7, the values for Load 1, Load 2 and Load 3, Load 9 and Load 10 decreased as the mass drawn increased. The maximum reduction rates of these loads were 11.71%, 9.81%, 5.44%, 4.97%, and 3.81%, respectively. Meanwhile, the peak value for Load 3 was higher than that in limiting equilibrium state.

Based on the above analysis, a new standpoint was proposed that the distribution laws were divided into two parts. One part was called the reductive region, which was located in the lower part of the bulk solids, and belonged to the area that was obviously influenced by ore drawing. The reductive region was smaller than the other part and close to the drawing hole. Thus, the values in the reductive region decreased as with the mass drawn increased in the beginning. Furthermore, the values slightly increased as the mass drawn continued to increase. The other part was not influenced by ore drawing, and was defined as the extensive region located in the upper part of the bulk solids. Meanwhile, its lower end coincided with the top of the reductive region, and its apex occurred on the top surface of the bulk solids. The friction coefficient between the bulk solids and collapse pit wall increased in the extensive region as the ore loading increased, and the values of lateral pressure were not influenced by ore drawing. As a result, the values in the extensive region increased with the increase of mass drawn.

Clearly, the dipping angle had a notable impact on the basic range and variation laws of the reductive region. Meanwhile, the dipping angle and the heights of bulk solids both had a primary influence on the maximum reduction rates of the reductive region. As the dipping angle decreased, the scope of reductive region and the maximum reduction rates decreased in the lower wall, while the scope of reductive region remained constant and the maximum reduction rates decreased in the upper wall. According to these results, the scope of reductive region in the lower wall was significantly influenced by the dipping angle. Likewise, the maximum reduction rates of the upper wall and lower wall decreased as the heights of bulk solids decreased. Meanwhile, the maximum reduction rates and the scope of reductive region in the lower wall were higher than those in the upper wall, indicating the reductive region of the lower wall was significantly influenced by ore drawing. The maximum growth rates of the extensive region in the upper wall were less than those in the lower wall.

### Experimental discussion

**Fig 8:**
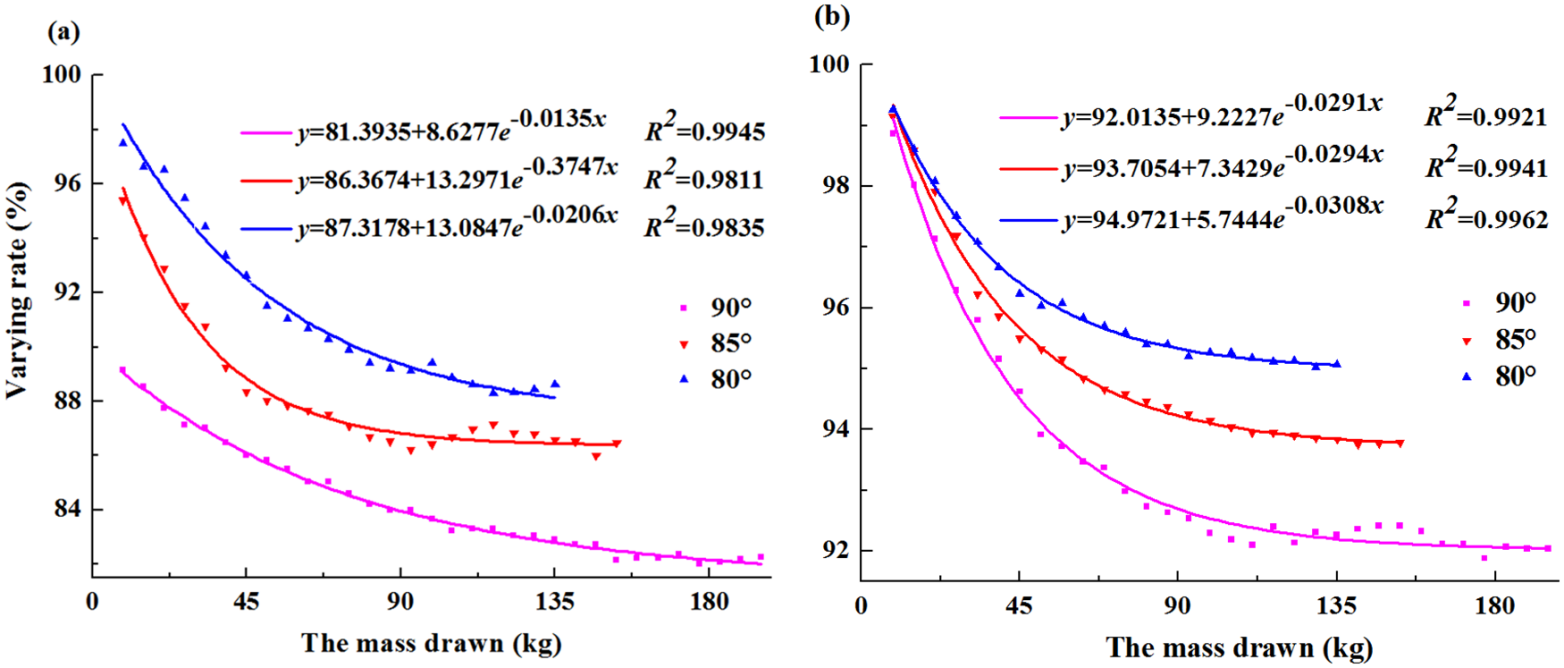
The relationships between the reduction rates of lateral pressure and the drawn mass with different angles. (a) The reduction rates of lateral pressure in Load 1 and (b) the reduction rates of lateral pressure in Load 9.

Based on the experimental analysis, the maximum reduction rates decreased as the depth of bulk solids decreased in the reductive region. Therefore, the variation laws of lateral pressure in Load 1and Load 9 were used to investigate the scope of CRZ. The relationships between the reduction rates of lateral pressure and the drawn mass with different angles were presented in Fig 8. Thus, the exponential relationships between the reduction rates and the drawn mass were obtained. In addition, the reduction rates and the values of lateral pressure decreased as the angle decreased in the above loads. The manner of the face-end ore drawing was often used to exploit orebody in the field application of caving mining method. In the other words, the dipping angle of the drawing hole was vertical to increase the mining efficiency and decrease the residual ore [27]. Hence, the modified formulas of the scope of CRZ could be summarized as follows:

$$L_{1}=\frac{1}{2}L_{t}+\text{cot}\beta \left[H-\frac{S}{fKC}\text{ln}\frac{S\gamma (81.3935+8.6277{\text{e}}^{-0.0404\text{x}})}{S\gamma (81.3935+8.6277{\text{e}}^{-0.0404\text{x}})-100fCp}\right]$$ (4)

$$L_{2}=\frac{1}{2}L_{t}+\text{cot}\beta \left[H-\frac{S}{fKC}\text{ln}\frac{S\gamma (92.0135+9.2227{\text{e}}^{-0.0871\text{x}})}{S\gamma (92.0135+9.2227{\text{e}}^{-0.0871\text{x}})-100fCp}\right]$$ (5)

where *L*_*1*_ is the distance between the scope of CRZ of the hanging wall and the stope centre, (m); *L*_*2*_ is the distance between the scope of CRZ of the footwall and the stope centre, (m); and $x=\frac{m}{300}$, where *m* is the mass of ore in a stope, (kg).

### Field test

Dabeishan Iron Mine is a typical example of many iron mines and is located in Liaoning Province, China (41.22 degrees north latitude and 123.41 degrees east longitude). This mine employs caving mining method with the sublevel of 40 m, and a series of parameters obtained in the field investigation are shown in Table 1 [28].

**Table 1:** The parameters for Dabeishan Iron Mine, China.

*H*(m)	*β*(°)	*p*(Pa)	*f*	*K*	*S/C*(m)	*ρ*(kg·m^-3^)
350	82	1.05×10^6^	0.7813	0.4718	57.25	1.81×10^3^

**Fig 9:**
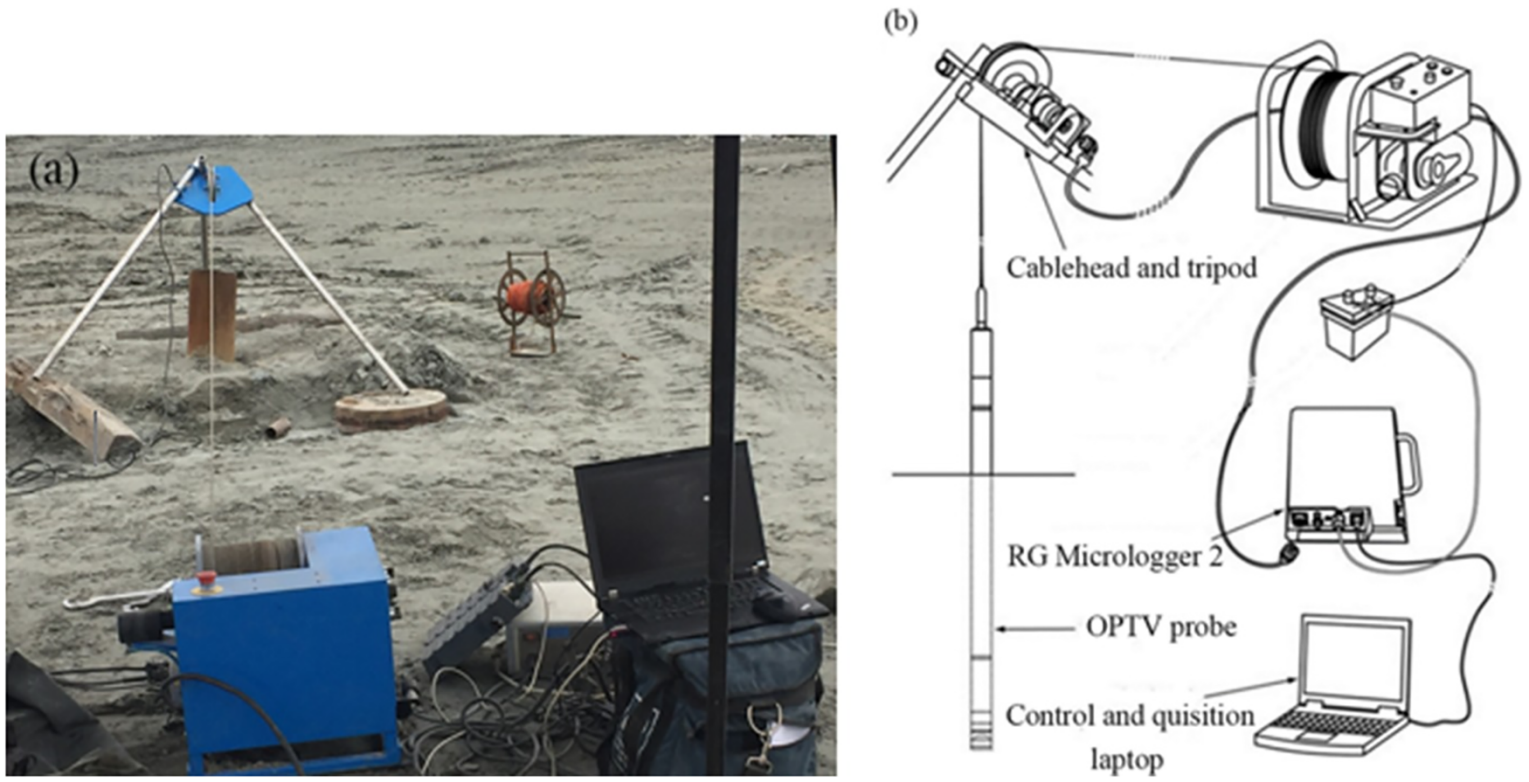
OPTV system components (a) in operation at Dabeishan Iron Mine, China and (b) illustrated as a line drawing.

Digital Optical Televiewing (OPTV) differed from traditional directional borehole video in that it records, a 360° annular image of the complete borehole circumference. These annuli are then stacked-guided by precise depth control to produce an orientated and geometrically accurate image of a complete borehole wall. The benefits of OPTV are related both to its complete coverage and to its ability to recreate the geometrical properties of any visibly identifiable object or layer that intersects a logged borehole [29–31]. The instrument was raised or lowered along the borehole by a winch, which, along with the OPTV probe, relayed dip, dip-direction and image data back to a data logger (RG Micrologger 2) located at the surface (Fig 9). Surface vertical boreholes are usually used to monitor rock failure, and their bottoms are required to enter the horizontal line of the gob roof. Bryn et al. conducted a field test to assess the numerous natural and drilling-related properties of different boreholes in Greenland [32].

**Fig 10:**
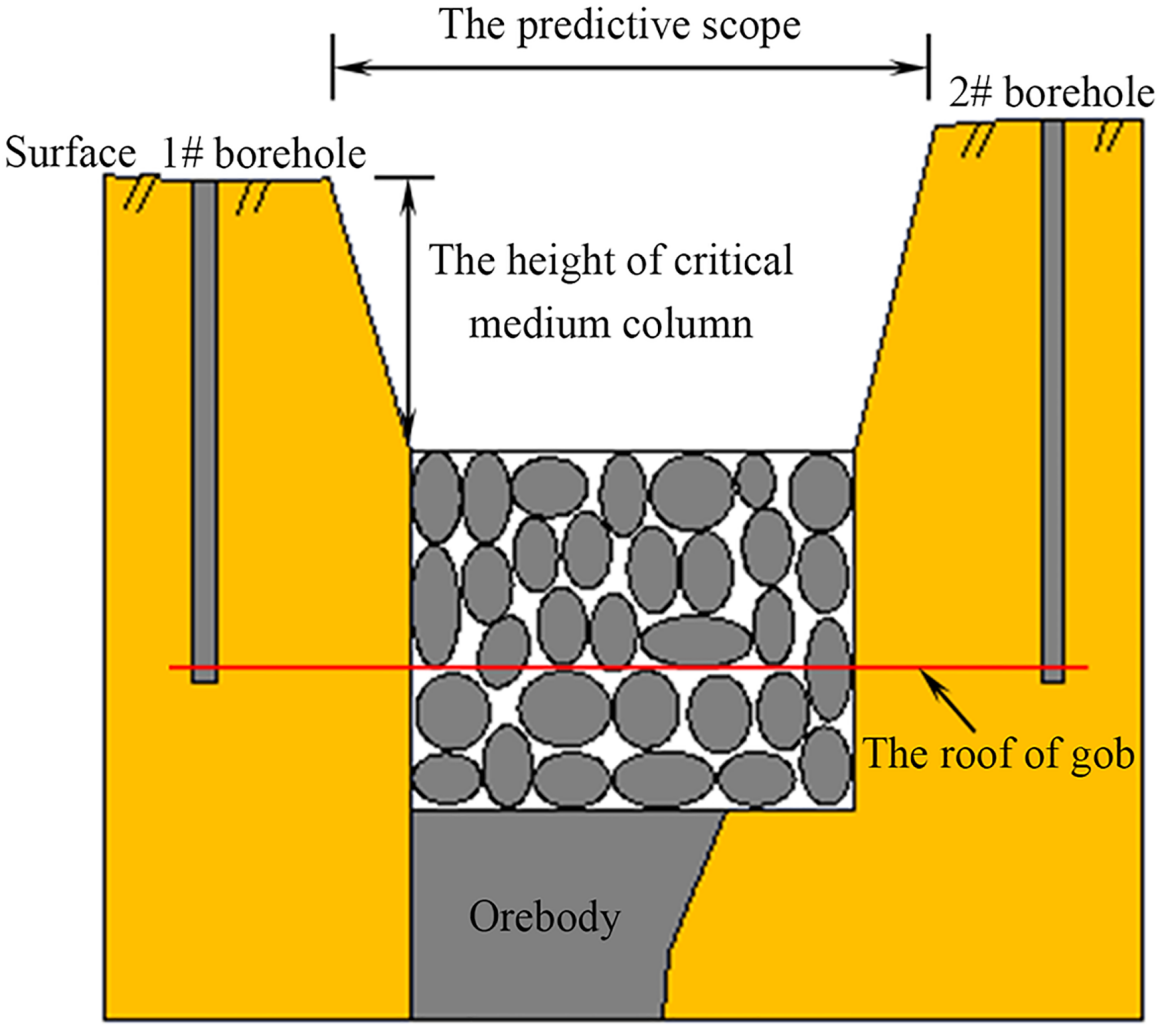
Simplified geological setting of Dabeishan Iron Mine, China.

**Fig 11:**
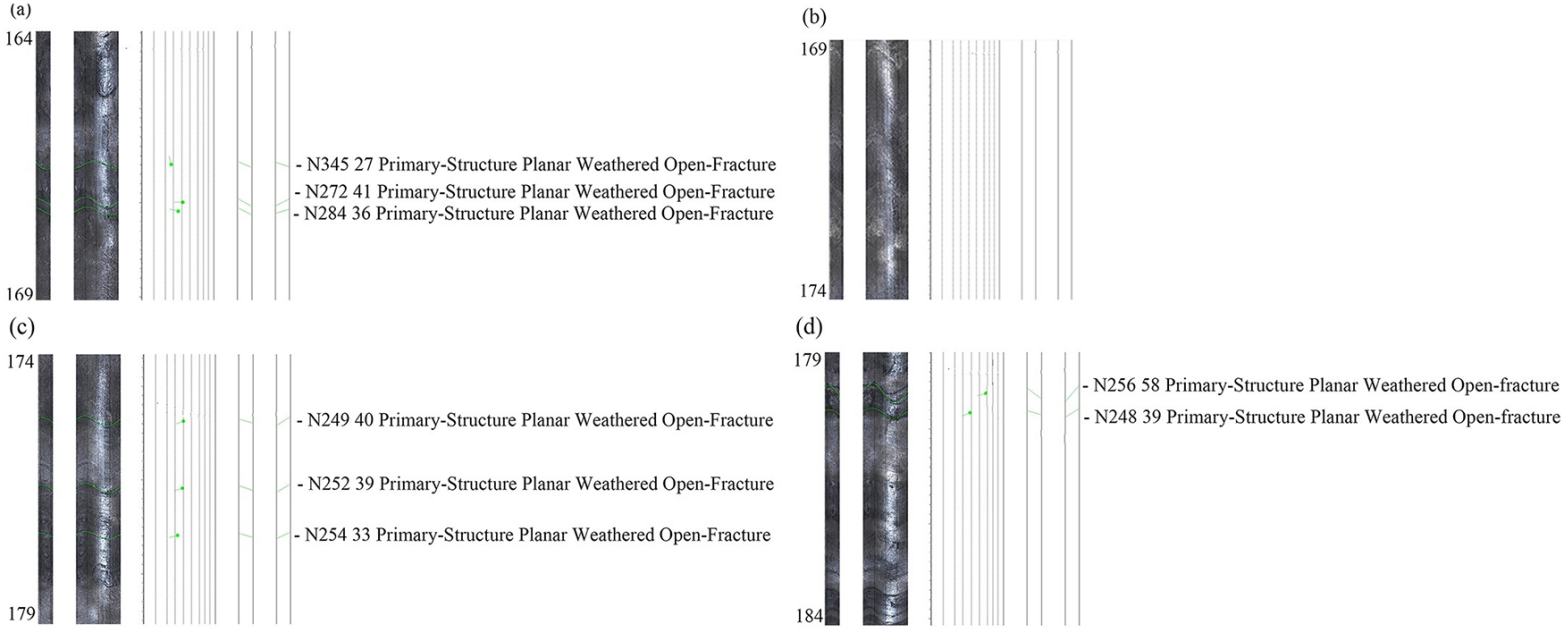
The characteristics of the rock and ore in the 1# borehole (a-d).

**Fig 12:**
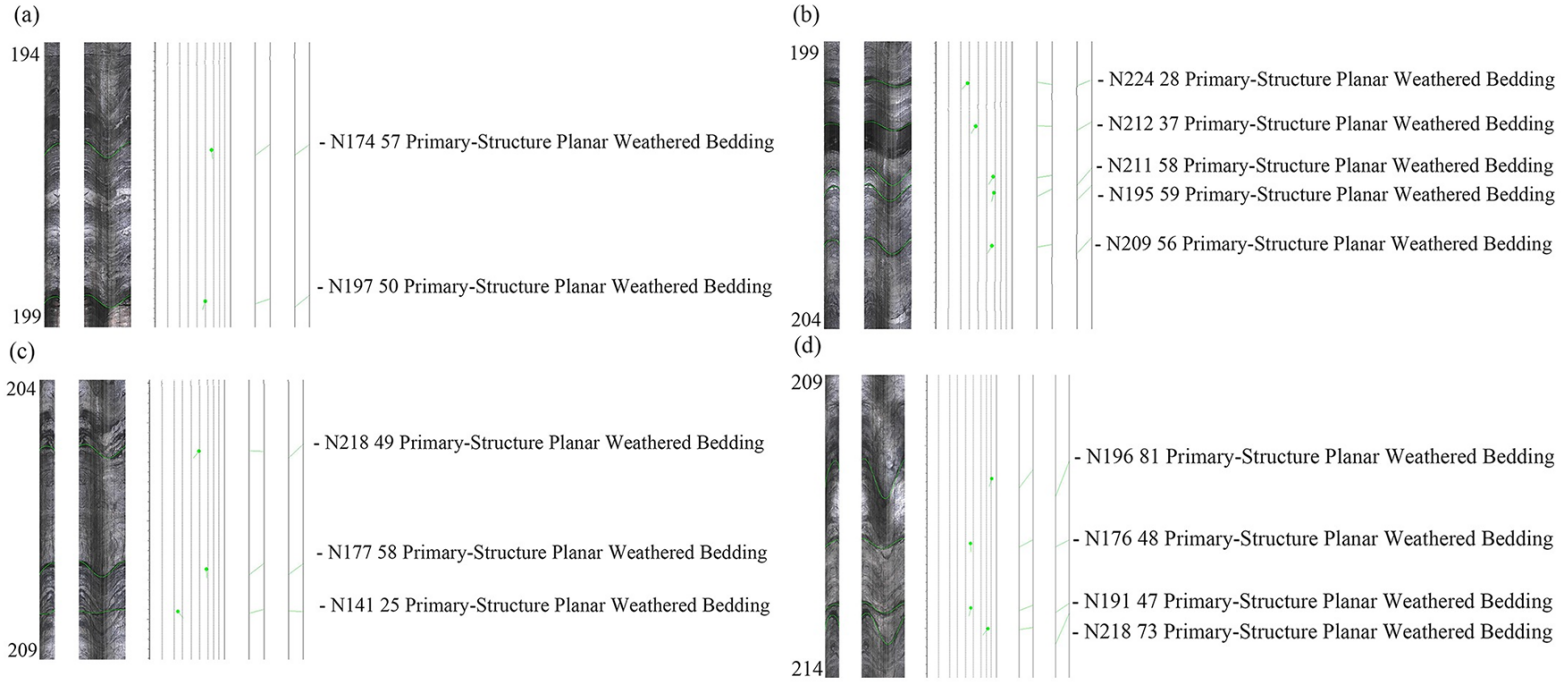
The characteristics of the rock and ore in the 2# borehole (a-d).

A simplified geological setting of the mine is shown in Fig 10. The lengths of the 1# and 2# boreholes were 184.0 m and 214.0 m, respectively. The distance between these two boreholes and the boundary of the predictive CRZs’ scope were 4.0 m. The bottoms of the two boreholes both entered the level of the gob roof, and their labels were 184.0 and 214.0 m, respectively. The properties and fractures of the rock mass for the 1# and 2# boreholes after mining the lower deposit were shown in Figs 11 and 12. Due to the two boreholes were large in volume and the rock mass failed more easily closer to the gob roof, the range from 164.0 to 184.0 m in the 1# borehole and that from 194.0 to 214.0 m in the 2# borehole were selected to analyse the failure of the rock mass.

As shown in Figs 11 and 12, the fractures of the rock mass were the primary-structure planar weathered bedding with the monitoring of OPTV. Hence, it could be clearly observed that the rock mass were not ruptured after mining the lower orebody. The field test indicated that the prediction method in this study was valid and could be used in practice.

## Conclusions

In this study, a laboratory-scaled physical model was designed to predict the scope of CRZ induced by caving mining method, which also was used to investigate to the influence of ore drawing and different dipping angles on the lateral pressure. The characteristics of lateral pressures’ variation laws and the predictive CRZs’ scope were obtained. Then the field test in Dabeishan Iron Mine was performed. According to this study, some conclusions could be made as follows:

1. Based on the comparison of the results between physical experiments and theoretical equation with different angles under the limiting equilibrium state, the reliability of the critical medium column theory is validated for predicting the scope of CRZ.
2. The distribution laws of lateral pressure were divided into two parts based on the experimental data. Meanwhile, the reductive region was smaller than the other part and close to the drawing hole. In addition, the depth of bulk solids decreased as the maximum reduction rates of the reductive region decreased.
3. The scope of reductive region and the maximum reduction rates on the lower wall were greater than those on the other side. Moreover, decreasing the dipping angle was an effective way of controlling the maximum reduction rates and the scope of reductive region. In addition, the predictive formula of CRZs’ scope induced by caving mining method was established based on the experimental analysis and the actual mining experience.
4. The field test of Dabeishan Iron Mine showed that the fractures of the rock mass were the primary-structure planar weathered bedding with the monitoring of OPTV. Hence, it could be clearly observed that the rock mass were not ruptured after mining the lower orebody. These results indicated that the predicted formula was valid and could be used in practice.

## Supporting information

S1 Fig The relationship between the depth of bulk solids and the lateral pressure in physical experiments and theoretical equation under the limiting equilibrium state. (PDF)

S2 Fig Depth of bulk solids and the lateral pressure with the dipping angle of 90°. (PDF)

S3 Fig Depth of bulk solids and the lateral pressure with the dipping angle of 85°. (PDF)

S4 Fig Depth of bulk solids and the lateral pressure with the dipping angle of 80°. (PDF)

S5 Fig The relationship between the reduction rates of lateral pressure and the drawn mass with different angles. (PDF)
