# Supplementary material for: Prediction of the caved rock zones’ scope induced by caving mining method
Source: PLoS One. 2018 Aug 15;13(8):e0202221. doi: 10.1371/journal.pone.0202221 (PMC6093666; doi:10.1371/journal.pone.0202221)
Supplement: S1 Fig — (PDF) [file pone.0202221.s001.pdf]

x	90°theoretical data	90°experimental data
0.065	318.3434209	243.4790054
0.195	857.5727546	726.2715374
0.325	1288.76021	1197.333736
0.455	1633.553437	1609.106846
0.585	1909.262686	1938.111284
0.715	2129.729863	2211.438274
0.845	2306.023465	2408.53554
0.975	2446.994264	2591.47853
	85°theoretical data	85°experimental data
	299.4936656	229.0763653
	806.4755872	682.9266117
	1211.530998	1125.183325
	1535.151779	1556.308723
	1793.710009	1825.368575
	2000.286231	2077.446506
	2165.331201	2285.634232
	2297.194598	2470.83763
	80°theoretical data	80°experimental data
	280.2430388	214.050035
	753.7325485	637.4601722
	1131.048712	1049.63459
	1431.725884	1405.281771
	1671.330679	1689.927586
	1862.267881	1932.582101
	2014.422662	2122.66357
	2135.672355	2306.872461
