# Supplementary material for: Prediction of the caved rock zones’ scope induced by caving mining method
Source: PLoS One. 2018 Aug 15;13(8):e0202221. doi: 10.1371/journal.pone.0202221 (PMC6093666; doi:10.1371/journal.pone.0202221)
Supplement: S2 Fig — (PDF) [file pone.0202221.s002.pdf]

(a)

The mass drawn (kg)	1	2	3	4
0	2591.4785	2408.5355	2211.4383	1938.1113
6	2310.369	2352.3647	2208.0361	1928.8904
12	2294.0546	2304.7047	2191.025	1925.065
18	2273.9753	2279.1725	2167.2095	1920.7578
24	2257.661	2246.8317	2139.9918	1911.8401
30	2255.151	2246.8317	2116.1763	1909.5349
36	2241.3466	2223.0017	2099.1653	1906.3553
42	2228.797	2200.8738	2094.0619	1906.5838
48	2223.7772	2187.2567	2073.6487	1918.1992
54	2216.2475	2185.5545	2061.7409	1908.2431
60	2203.698	2165.1288	2063.442	1886.6717
66	2203.698	2168.5331	2053.2354	1871.7376
72	2192.4034	2151.5116	2044.7298	1825.276
78	2182.3638	2149.8095	2037.9254	1806.934
84	2176.089	2143.0009	2026.0177	1798.7266
90	2176.089	2141.2987	2026.0177	1855.1442
96	2168.5593	2125.9794	2019.2133	1835.2321
102	2157.2647	2124.2773	2014.1099	1803.6153
108	2158.5196	2124.2773	1993.6967	1843.5288
114	2158.5196	2120.873	1997.0989	1841.8695
120	2152.2449	2107.2558	1998.8	1825.276
126	2152.2449	2112.3623	1981.7889	1816.9793
132	2148.48	2112.3623	1981.7889	1814.9524
138	2143.4602	2098.7451	1974.9845	1808.6826
144	2143.4602	2090.2344	1957.9734	1835.2321
150	2128.4008	2095.3408	1961.3756	1838.5508
156	2130.9107	2095.3408	1957.9734	1831.9134
162	2130.9107	2080.0215	1971.5823	1845.1881
168	2134.6755	2080.0215	1963.0767	1830.2541
174	2124.6359	2080.0215	1976.6856	1833.5727
180	2127.1458	2078.3194	1983.49	1840.2101
186	2129.6557	2086.8301	1974.9845	1843.5288
192	2132.1656	2085.1279	1985.1911	1850.1662

(b)

The mass drawn (kg)	5	6	7	8
0	1609.1068	1197.3337	726.27154	243.47901
6	1720.1294	1278.6953	821.16898	270.46704
12	1676.8739	1300.6849	834.17911	300.97525
18	1672.5483	1260.0041	843.36273	327.37659
24	1705.7109	1283.0932	832.64851	342.6307
30	1723.0132	1311.6797	838.00562	370.79212
36	1747.5246	1294.088	845.65864	381.35266
42	1796.5476	1260.0041	875.50541	387.21962
48	1754.7339	1369.9521	871.67891	371.96552
54	1767.7106	1329.2713	916.06642	373.13891
60	1763.385	1375.4495	913.00521	387.21962
66	1796.5476	1397.4391	943.61729	362.57837
72	1776.3617	1421.6277	988.0048	355.53802
78	1748.9665	1468.9053	1023.2087	340.87061
84	1731.6643	1507.3871	1020.1475	340.87061
90	1751.8502	1478.8006	1058.4126	346.73757
96	1774.9198	1485.3975	1088.2594	335.00364
102	1772.0361	1482.0991	1046.1677	352.60454
108	1756.1757	1482.0991	1062.2391	342.6307
114	1797.9895	1455.7115	1095.9124	363.16507
120	1831.1521	1433.7219	1098.9736	352.60454
126	1864.3147	1412.8318	1103.5654	357.8848
132	1844.1287	1442.5178	1109.6878	360.23159
138	1896.0354	1442.5178	1096.6777	356.12471
144	1888.8261	1451.3136	1079.841	379.00588
150	1877.2913	1473.3032	1105.8613	379.00588
156	1878.7332	1494.1933	1090.5553	381.93935
162	1958.035	1472.2037	1043.1065	373.13891
168	1921.9887	1479.9001	1062.2391	373.13891
174	1943.6165	1466.7063	1060.7085	391.49488
180	1954.4537	1453.5126	1064.6063	400.54526
186	1942.1747	1461.2089	1071.7016	401.88703
192	1965.2443	1476.2696	1076.7441	405.11106

(c)

The mass drawn (kg)	9	10	11	12
0	2591.4785	2408.5355	2211.4383	1938.1113
6	2562.1314	2398.0408	2266.0966	2029.1607
12	2540.3214	2388.858	2280.6543	2068.4968
18	2517.2354	2363.933	2311.3835	2106.6249
24	2495.3858	2348.191	2342.4571	2143.545
30	2482.6262	2339.0081	2351.721	2167.3883
36	2465.9596	2329.8252	2350.3976	2192.5452
42	2452.0797	2332.4489	2339.8102	2179.2571
48	2433.7441	2320.6424	2330.5463	2180.4651
54	2428.6403	2319.3305	2337.1634	2191.3372
60	2422.2605	2310.1477	2325.2526	2170.801
66	2419.7085	2311.4595	2327.8994	2162.3449
72	2409.5008	2304.9003	2318.6355	2144.2247
78	2403.121	2299.6529	2321.2823	2145.4327
84	2400.5691	2291.7819	2297.4607	2115.2324
90	2398.0172	2286.5346	2293.4904	2126.1045
96	2391.6374	2282.599	2289.5202	2118.8564
102	2389.0855	2278.6635	2288.1968	2124.8965
108	2386.5335	2274.728	2282.9031	2120.0644
114	2394.5058	2276.0398	2286.8733	2123.6885
120	2387.6666	2270.7925	2281.5796	2132.1445
126	2392.0329	2272.1043	2284.2265	2138.1846
132	2390.8756	2268.1688	2293.4904	2140.6006
138	2393.5462	2264.2333	2286.8733	2134.5606
144	2394.9013	2256.3623	2280.2562	2132.1445
150	2394.9409	2255.0504	2286.8733	2144.2247
156	2392.4285	2252.4268	2277.6094	2124.8965
162	2387.3246	2247.1794	2280.2562	2133.3526
168	2387.3246	2247.1794	2284.2265	2139.3926
174	2380.9448	2244.5557	2286.8733	2141.8087
180	2385.8703	2248.275	2290.8436	2150.2648
186	2385.0851	2251.0313	2291.4483	2152.3018
192	2385.0864	2252.3921	2295.3325	2157.3635

(d)

The mass drawn (kg)	13	14	15	16
0	1609.1068	1197.3337	726.27154	243.47901
6	1736.9441	1231.476	723.2766	265.48841
12	1742.8081	1239.7172	754.72341	265.48841
18	1772.1286	1266.7956	771.19555	264.11282
24	1773.3014	1302.1152	795.15502	279.24428
30	1773.3014	1315.0657	883.50558	276.49311
36	1795.585	1326.8389	902.97265	284.74663
42	1803.7947	1336.2574	943.40427	290.24898
48	1806.1403	1329.1935	989.82575	290.24898
54	1820.2142	1342.144	974.85107	283.37104
60	1821.387	1359.8038	1034.7498	277.86869
66	1816.6957	1385.7048	1069.1915	299.8781
72	1829.5967	1401.01	1079.6738	309.50721
78	1854.2259	1418.6698	1102.1358	320.51191
84	1841.3249	1415.1378	1171.0193	319.13632
90	1882.3735	1423.379	1169.5218	339.77014
96	1854.2259	1408.0739	1214.4458	346.64807
102	1843.6705	1396.3007	1257.8724	326.01426
108	1823.7326	1413.9605	1253.3799	308.13162
114	1820.2142	1455.1667	1293.8116	337.01896
120	1812.0044	1455.1667	1292.3141	349.39925
126	1835.4608	1458.6986	1277.3394	364.90979
132	1830.7695	1483.4223	1307.2888	370.41214
138	1821.387	1488.1316	1308.7862	370.41214
144	1844.8433	1523.4512	1275.842	363.5342
150	1849.5346	1535.2244	1281.8318	358.03185
156	1837.8064	1556.4161	1292.3141	339.95967
162	1855.3987	1515.2099	1257.8724	349.58879
168	1848.3618	1531.6924	1230.9179	360.59349
174	1875.3366	1525.8058	1253.3799	359.2179
180	1892.9289	1556.4161	1269.8521	339.77014
186	1901.6793	1537.579	1293.8116	346.64807
192	1906.9112	1559.9481	1304.2938	358.07952
