# Supplementary material for: Prediction of the caved rock zones’ scope induced by caving mining method
Source: PLoS One. 2018 Aug 15;13(8):e0202221. doi: 10.1371/journal.pone.0202221 (PMC6093666; doi:10.1371/journal.pone.0202221)
Supplement: S3 Fig — (PDF) [file pone.0202221.s003.pdf]

(a)

The mass drawn (kg)	1	2	3	4
0	2470.8376	2285.6342	2077.4465	1825.3686
6	2357.2359	2213.3422	2071.9007	1884.2102
12	2323.8982	2104.6813	2023.0979	1880.7011
18	2295.2713	2078.397	2027.5346	1884.7495
24	2261.7072	2085.6262	2043.0627	1875.2485
30	2242.3432	2083.2164	2051.936	1874.0609
36	2204.9063	2077.1921	2018.6613	1881.1866
42	2182.9605	2072.3726	1995.3691	1869.796
48	2175.2149	2069.9629	1975.4043	1857.9198
54	2170.0512	2063.9386	1963.2036	1866.2331
60	2166.1784	2066.3483	1949.8937	1848.4188
66	2162.3057	2061.5288	1953.2212	1849.6065
72	2151.9782	2063.9386	1925.4923	1832.2777
78	2141.6508	2055.5045	1911.0733	1811.6027
84	2137.778	2036.2266	1908.855	1806.4743
90	2130.0324	2037.4315	1904.4184	1814.5986
96	2135.1962	2037.4315	1902.2001	1829.4169
102	2141.6508	2036.2266	1898.8726	1816.3531
108	2149.3964	2061.5288	1899.9818	1812.7903
114	2153.2692	2051.8899	1897.7635	1812.7903
120	2145.5236	2054.2996	1897.7635	1814.0186
126	2144.2327	2062.7337	1902.2001	1815.6787
132	2139.0689	2051.8899	1905.5275	1812.1158
138	2137.778	2060.324	1907.7459	1820.6181
144	2124.8687	2057.9142	1912.1825	1828.5535
150	2136.4871	2062.7337	1918.8374	1837.8655

(b)

The mass drawn (kg)	5	6	7	8
0	1556.3084	1125.1833	682.92661	229.07637
6	1630.2559	1179.3328	727.01288	253.58602
12	1729.1406	1258.0956	768.12285	284.89312
18	1699.9639	1227.8839	807.23592	350.56168
24	1682.6789	1201.9906	831.92878	373.67594
30	1664.1442	1228.2217	864.83342	397.90413
36	1643.9714	1242.8166	899.24851	377.3699
42	1634.1238	1262.9403	939.32159	384.86022
48	1673.4411	1302.5624	969.0516	394.16103
54	1643.9531	1372.0204	942.48976	400.19489
60	1678.3558	1409.9954	970.29329	409.97782
66	1640.6767	1448.6735	985.95518	397.20345
72	1661.9736	1427.9504	1016.2356	388.66623
78	1677.5366	1437.0858	1022.6553	387.82781
84	1659.5162	1456.4092	1033.76	393.66101
90	1742.2463	1475.8572	1038.1893	403.03665
96	1702.1099	1507.7457	1060.13	397.3412
102	1710.301	1483.0039	1068.201	401.57835
108	1744.7036	1477.5064	1065.9993	396.99384
114	1752.0756	1460.4935	1069.2443	397.55379
120	1766.8196	1459.6551	1070.6139	400.53927
126	1757.8094	1461.2243	1097.8606	393.46035
132	1749.9382	1467.7961	1070.3322	396.87285
138	1759.0021	1476.4254	1052.9487	401.03332
144	1763.9264	1478.2096	1063.7847	403.46779
150	1770.8014	1484.3237	1065.7816	409.22616

(c)

The mass drawn (kg)	9	10	11	12
0	2470.8376	2285.6342	2077.4465	1825.3686
6	2449.8528	2276.849	2136.9744	1890.6962
12	2435.909	2263.6711	2188.6586	1950.6972
18	2419.4604	2259.2784	2226.7566	1992.447
24	2401.44	2254.8858	2251.1239	2037.2015
30	2377.5208	2253.4216	2287.2414	2079.5191
36	2368.5993	2243.1721	2271.7199	2113.5056
42	2359.6036	2238.7795	2257.4565	2106.921
48	2355.2337	2232.9226	2253.2006	2099.5386
54	2351.2628	2228.53	2251.7017	2092.8247
60	2343.3348	2219.7447	2244.3999	2083.329
66	2338.9104	2216.8163	2236.7129	2074.7641
72	2336.8364	2212.4237	2232.0718	2088.5046
78	2334.1067	2202.1742	2228.8815	2091.5956
84	2331.913	2199.2458	2231.7829	2090.0645
90	2328.765	2199.2458	2233.5226	2090.1939
96	2326.123	2196.903	2234.6362	2093.6443
102	2323.4566	2193.3889	2242.4195	2086.8011
108	2321.163	2183.1394	2243.8702	2094.09
114	2321.138	2175.8184	2249.7213	2089.8058
120	2320.1978	2169.9615	2249.5768	2094.8879
126	2318.9783	2178.7468	2255.3797	2088.5263
132	2318.532	2172.8899	2253.9772	2089.5758
138	2316.625	2177.2826	2256.9268	2092.1096
144	2317.0062	2179.2568	2258.3775	2092.7063
150	2317.1463	2180.9349	2259.8956	2094.4494

(d)

The mass drawn (kg)	13	14	15	16
0	1556.3087	1125.1833	682.92661	229.07637
6	1623.0331	1153.32	703.96948	253.78668
12	1667.8524	1177.9715	730.26064	256.84103
18	1679.171	1210.2618	747.18464	272.65782
24	1664.7952	1228.8962	793.14739	275.01746
30	1678.4686	1252.7511	777.17048	274.185
36	1691.1298	1253.9742	797.65557	279.7427
42	1711.6081	1282.3241	843.79623	270.03461
48	1712.6717	1293.3908	851.27697	265.45905
54	1727.7797	1277.1746	851.75323	268.38161
60	1715.0463	1268.9556	893.91675	281.0242
66	1715.531	1269.3797	933.23087	297.54167
72	1706.4435	1259.1948	968.86348	293.6087
78	1706.5573	1276.2076	964.29237	294.37342
84	1705.5036	1275.9374	994.8174	287.94378
90	1704.5538	1283.5761	1032.763	316.42474
96	1710.0993	1298.555	1052.6595	317.55059
102	1727.7797	1287.8578	1114.5992	319.03531
108	1717.8759	1305.5392	1127.6035	325.16858
114	1713.859	1306.6062	1147.0238	325.59376
120	1736.4962	1309.6788	1123.1014	322.86404
126	1746.6276	1308.0855	1099.6295	318.75683
132	1752.5243	1320.4042	1106.4615	327.34431
138	1753.9238	1326.4355	1107.8274	331.90127
144	1758.6585	1330.3048	1112.1604	334.46271
150	1765.3715	1340.2195	1115.093	338.92211
