# Supplementary material for: Prediction of the caved rock zones’ scope induced by caving mining method
Source: PLoS One. 2018 Aug 15;13(8):e0202221. doi: 10.1371/journal.pone.0202221 (PMC6093666; doi:10.1371/journal.pone.0202221)
Supplement: S4 Fig — (PDF) [file pone.0202221.s004.pdf]

(a)

The mass drawn (kg)	1	2	3	4
0	2306.8725	2122.6636	1932.5821	1689.9276
6	2249.482	2078.0959	1923.9061	1729.6119
12	2229.2265	2070.6573	1917.3991	1733.5207
18	2226.9759	2068.112	1909.8076	1732.6901
24	2202.2192	2069.8043	1907.6386	1732.6901
30	2178.5878	2047.0022	1900.0471	1735.9545
36	2153.8312	2045.9658	1900.0471	1725.9961
42	2136.9516	2024.2002	1894.6245	1722.1245
48	2111.0696	2022.1273	1889.202	1723.4994
54	2099.8166	2032.4918	1885.9485	1718.8067
60	2091.9395	2024.3038	1881.6105	1711.8359
66	2082.937	2002.4346	1872.9345	1712.8317
72	2073.9346	2001.3981	1866.4275	1720.3191
78	2062.6816	1982.7419	1857.7515	1729.2816
84	2058.1804	1968.2315	1845.822	1739.7937
90	2055.9297	1966.1586	1842.5685	1735.0169
96	2062.6816	1962.0128	1838.2305	1734.5376
102	2050.3032	1952.6847	1838.2305	1718.2157
108	2044.6767	1959.9398	1837.1459	1722.6411
114	2036.7996	1937.1378	1830.6389	1734.149
120	2037.9249	1936.1013	1827.3854	1742.9091
126	2040.1755	1914.3357	1839.3149	1744.1446
132	2044.6767	1924.7003	1841.484	1749.3634

(b)

The mass drawn (kg)	5	6	7	8
0	1405.2818	1049.6346	637.46017	214.05004
6	1516.9026	1118.1609	684.35524	234.03374
12	1494.5032	1168.2192	716.94007	259.07815
18	1478.8751	1154.2021	727.37223	281.41654
24	1458.7245	1133.6338	720.42465	303.32381
30	1477.1229	1149.5063	726.48376	310.27002
36	1503.69	1168.8397	751.79891	326.12773
42	1565.8691	1191.2633	775.71119	322.26707
48	1586.7077	1229.4264	772.74102	333.97059
54	1594.5788	1280.0256	809.71834	337.17654
60	1606.7873	1290.6737	823.44487	331.7645
66	1588.7407	1308.4555	835.65821	326.61046
72	1576.5461	1380.9953	886.94577	326.49006
78	1570.1048	1409.8827	888.64917	315.6488
84	1584.3879	1404.3299	901.29491	312.99438
90	1586.6367	1391.9556	924.56302	323.76685
96	1596.5577	1389.7719	942.34051	338.74511
102	1578.7517	1373.9667	939.69031	340.58887
108	1570.1078	1363.2769	934.10459	334.26298
114	1562.5313	1349.8254	933.55553	325.16228
120	1576.0524	1336.9271	936.86723	326.54166
126	1586.3991	1350.986	942.45295	330.50552
132	1597.6467	1358.265	949.21033	337.04915

(c)

The mass drawn (kg)	9	10	11	12
0	2306.8725	2122.6636	1932.5821	1689.9276
6	2289.8182	2118.691	1998.1	1750.4742
12	2275.0377	2113.3943	2063.0255	1807.6817
18	2262.5806	2113.3943	2115.376	1847.199
24	2249.4644	2106.7734	2168.2883	1884.9352
30	2239.5513	2110.7459	2198.3237	1941.7797
36	2230.1033	2102.8008	2166.2064	1982.076
42	2219.8707	2094.8558	2161.1093	2019.6912
48	2215.3559	2086.9107	2162.7739	2034.5641
54	2216.5918	2088.2348	2159.5478	2027.5909
60	2210.973	2085.5865	2150.9464	2026.3316
66	2207.8423	2073.6688	2141.0618	2023.8131
72	2205.2882	2067.0479	2136.171	2020.8143
78	2200.8064	2060.5594	2133.4654	2019.5097
84	2200.8064	2055.1303	2128.0901	2019.1656
90	2196.2586	2052.8792	2130.4143	2012.9146
96	2197.5285	2048.5094	2131.038	2009.0461
102	2197.5285	2049.8336	2127.2914	2008.5205
108	2195.5923	2048.5094	2132.8058	2021.1131
114	2194.1165	2041.8885	2134.8518	2024.0211
120	2194.4428	2048.5094	2131.3475	2025.5338
126	2192.211	2047.1852	2133.6305	2028.7519
132	2192.9958	2053.8061	2135.1354	2030.0527

(d)

The mass drawn (kg)	13	14	15	16
0	1405.2818	1049.6346	637.46054	214.05004
6	1462.712	1095.6893	667.80338	234.03374
12	1464.1745	1102.132	691.55305	259.07815
18	1504.024	1121.6618	705.85903	281.41654
24	1516.5133	1126.2843	749.56624	303.32381
30	1518.8574	1141.5369	759.2651	310.27002
36	1526.6585	1169.4071	797.5383	326.12773
42	1538.3414	1194.5343	815.0904	322.26707
48	1551.2339	1212.8806	865.23845	333.97059
54	1552.734	1220.1202	906.24746	337.17654
60	1578.9222	1258.5486	925.09725	331.7645
66	1602.6398	1242.2257	942.03811	326.61046
72	1588.5377	1260.8508	990.08539	326.49006
78	1575.3699	1278.3877	1048.5694	315.6488
84	1592.1052	1263.9085	1090.6723	312.99438
90	1608.8419	1243.7905	1101.6689	323.76685
96	1617.4911	1263.4859	1085.7491	338.74511
102	1608.6013	1278.0479	1075.7856	340.58887
108	1607.1039	1289.6061	1066.1874	334.26298
114	1619.311	1294.9536	1072.5066	325.16228
120	1623.4292	1287.6366	1078.3129	326.54166
126	1636.6122	1301.5834	1081.5497	330.50552
132	1644.8985	1310.2745	1086.3277	337.04915
