# Supplementary material for: Prediction of the caved rock zones’ scope induced by caving mining method
Source: PLoS One. 2018 Aug 15;13(8):e0202221. doi: 10.1371/journal.pone.0202221 (PMC6093666; doi:10.1371/journal.pone.0202221)
Supplement: S5 Fig — (PDF) [file pone.0202221.s005.pdf]

90,1	85,1	80,1	90,9	85,9	80,9
100	100	100	100	100	100
89.15254	95.4023	97.5122	98.86755	99.1507	99.26072
88.523	94.05305	96.63415	98.02595	98.58637	98.62
87.74818	92.89446	96.53659	97.13511	97.92065	98.08
87.11864	91.53605	95.46341	96.29197	97.19133	97.51143
87.02179	90.75235	94.43902	95.79961	96.22327	97.08172
86.4891	89.2372	93.36585	95.15647	95.8622	96.67215
86.00484	88.34901	92.63415	94.62088	95.49813	96.22859
85.81114	88.03553	91.5122	93.91334	95.32126	96.03287
85.52058	87.82654	91.02439	93.7164	95.16056	96.08645
85.03632	87.6698	90.68293	93.47021	94.83969	95.84288
85.03632	87.51306	90.29268	93.37174	94.66063	95.70717
84.60048	87.09509	89.90244	92.97784	94.57669	95.59645
84.21308	86.67712	89.41463	92.73166	94.46621	95.40217
83.97094	86.52038	89.21951	92.63319	94.37743	95.40217
83.97094	86.2069	89.12195	92.53471	94.25002	95.20503
83.68039	86.41588	89.41463	92.28853	94.14309	95.26008
83.24455	86.67712	88.87805	92.19005	94.03518	95.26008
83.29298	86.9906	88.63415	92.09158	93.94235	95.17615
83.29298	87.14734	88.29268	92.39921	93.94134	95.11217
83.05085	86.83386	88.34146	92.1353	93.90329	95.12632
83.05085	86.78161	88.43902	92.30379	93.85393	95.02957
82.90557	86.57262	88.63415	92.25913	93.83587	95.06359
82.71186	86.52038		92.36219	93.75869	
82.71186	85.99791		92.41448	93.77412	
82.13075	86.46813		92.416	93.77979	
82.2276			92.31905		
82.2276			92.12211		
82.37288			92.12211		
81.98547			91.87592		
82.08232			92.06599		
82.17918			92.03569		
82.27603			92.03574		
